# The quantitative genetic basis of sex ratio variation in *Nasonia vitripennis*: a QTL study

**DOI:** 10.1111/j.1420-9101.2010.02129.x

**Published:** 2011-01

**Authors:** B A Pannebakker, R Watt, S A Knott, S A West, D M Shuker

**Affiliations:** * Institute of Evolutionary Biology, School of Biological Sciences, University of EdinburghEdinburgh, UK; † Centre for Ecological and Evolutionary Studies, University of GroningenGroningen, The Netherlands; ‡ Department of Zoology, University of OxfordOxford, UK; § School of Biology, University of St AndrewsSt Andrews, Fife, UK

**Keywords:** brood size, clutch size, linkage map, local mate competition, *Nasonia*, oviposition, parasitoid wasp, sex allocation

## Abstract

Our understanding of how natural selection should shape sex allocation is perhaps more developed than for any other trait. However, this understanding is not matched by our knowledge of the genetic basis of sex allocation. Here, we examine the genetic basis of sex ratio variation in the parasitoid wasp *Nasonia vitripennis*, a species well known for its response to local mate competition (LMC). We identified a quantitative trait locus (QTL) for sex ratio on chromosome 2 and three weaker QTL on chromosomes 3 and 5. We tested predictions that genes associated with sex ratio should be pleiotropic for other traits by seeing if sex ratio QTL co-occurred with clutch size QTL. We found one clutch size QTL on chromosome 1, and six weaker QTL across chromosomes 2, 3 and 5, with some overlap to regions associated with sex ratio. The results suggest rather limited scope for pleiotropy between these traits.

## Introduction

The study of sex allocation and sex ratios has produced many of the best tests of evolutionary theory to date (Charnov, 1982; Herre, 1987; West, 2009). This is in part attributed to a rigorous and well-developed theory base, originating from the ideas of frequency-dependent selection on sex allocation proposed by Düsing and Fisher (Fisher, 1930; Edwards, 2000) through to the ground-breaking work of Hamilton and Trivers (e.g. Hamilton, 1967; Trivers & Willard, 1973) and the many refinements of sex allocation theory developed by later evolutionary ecologists (reviewed by Charnov, 1982; Frank, 1998; Hardy, 2002; West, 2009). It is also partly attributed to the simplicity of the sex allocation trait itself. When considered in terms of the proportion of offspring that are male (sex ratio), the underlying trade-off is simply the production of either sons or daughters. As a result, patterns of sex ratios have been successfully predicted across an extremely broad range of organisms, from single-celled organisms such as *Plasmodium*, through to many plants and animals, largely regardless of the genetic system of sex determination (reviewed by West *et al.*, 2005; West, 2009). In contrast, we know much less about the genetic basis of sex ratio, and as such we have only half of the evolutionary story (Pannebakker *et al.*, 2008). For instance, understanding how sex ratios should evolve under natural selection is of limited use if genetic architecture constrains how sex ratio behaviour actually can evolve.

The genetic basis of sex ratio behaviour is important for three reasons. First, for all the successes of sex allocation theory, there still remains variation around predicted sex ratios (West & Herre, 1998; Orzack, 2002). To know how biologically meaningful this variation is we need to ascertain whether it is environmental or genetic in origin, and if the latter is the case, we need to assess whether variation around (or away from) the predicted optimum sex ratio represents a genetic constraint. Genetic constraints could arise through a lack of appropriate mutations (Blows & Hoffmann, 2005; but see Pannebakker *et al.*, 2008) or through particular genetic architectures, for instance if there are negative genetic correlations between fitness traits including sex ratio (Barton & Partridge, 2000). Second, identifying the genetic basis of sex ratio would allow us to explore the genetics of a well-characterized adaptation, including the molecular evolution of the genes associated with the trait. Third, that facultative sex allocation remains controversial in some taxa (particularly vertebrates with chromosomal sex determination) is not least because the proximate mechanisms underlying sex allocation are somewhat obscure (Krackow, 2002; West & Sheldon, 2002; Pike & Petrie, 2003; West *et al.*, 2005; Uller & Badyaev, 2009). Identifying the genetic basis of sex ratio traits in other taxa, such as parasitoid wasps, may provide another starting point for exploring sex allocation in species with less understood mechanisms of sex allocation.

To date, studies of sex ratio genetics have focused on quantitative genetic analyses, including studies on *Drosophila* (Toro & Charlesworth, 1982; Carvalho *et al.*, 1998), fish (Vandeputte *et al.*, 2007), turtles (Janzen, 1992), pigs (Toro *et al.*, 2006) and humans (Gellatly, 2009). However, an important group for sex ratio studies has been the parasitoid wasps. Genetic variation for sex ratio has been found in both *Trichogramma* and *Heterospilus* (Wajnberg, 1994; Kobayashi *et al.*, 2003), although most work has been carried out in *Nasonia vitripennis*, a species well known for its facultative sex ratio behaviour. Work by Orzack and colleagues in the 1980s and 1990s showed additive genetic variation in *N*. *vitripennis* sex ratios, using a mixture of artificial selection and quantitative genetic techniques (Parker & Orzack, 1985; Orzack & Parker, 1986, 1990; Orzack *et al.*, 1991; Orzack & Gladstone, 1994). More recently, we have explored how mutation inputs genetic variation in sex ratio, through the first mutation accumulation study of sex ratio (Pannebakker *et al.*, 2008). We found that in *N*. *vitripennis* one mutation occurs every 5–60 generations, which shifts the sex ratio by approximately 1% and that mutational heritabilities for sex ratio in *N*. *vitripennis* (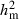 = 0.0008–0.0019) were similar to those for other life-history traits in species such as *Drosophila melanogaster* and *Caenorhabditis elegans*. Moreover, by estimating the strength of stabilizing selection on sex ratio in *Nasonia*, we estimated the degree of standing genetic variation for sex ratio we would expect to see in a population and compared this to our own estimate of sex ratio heritability and those of Orzack and colleagues. We predicted greater genetic variation in sex ratio than any study has so far observed, suggesting that sex ratio genes (i.e. genes that influence variation for sex allocation) are pleiotropic, influencing other fitness-related traits (and thus facing stronger purifying selection, leading to less variation at mutation-selection balance: Turelli, 1984).

In this study, we consider the genetic basis of sex ratio in *N*. *vitripennis* using a quantitative trait locus (QTL) approach. We chose two wasp strains that diverged in their patterns of sex ratio (specifically in their sex ratios when a single female lays eggs on a host) and performed a QTL study to identify regions of the genome associated with this intra-specific variation. Given that our previous work made the prediction that at least some sex ratio genes should be pleiotropic, influencing other fitness-related traits in *N*. *vitripennis*, we also looked for QTL for clutch size in crosses between the same two lines to see whether or not sex ratio and clutch size QTL overlapped. Theory suggests that clutch size and sex ratio should be simultaneously optimized by natural selection when females lay eggs across multiple patches and kin-related processes such as local mate competition (LMC) influence sex ratio (Werren, 1980; Godfray, 1986; Nagelkerke, 1994; Greeff, 1997; West *et al.*, 1997). More practically, clutch size is a straightforward trait to analyse alongside sex ratio. Whilst the prediction of pleiotropic gene action can only be confirmed once individual genes are identified and cloned, we could at least ascertain whether such pleiotropy was plausible.

## Materials and methods

### Study organism

The parasitoid wasp *N. vitripennis* (Hymenoptera: Chalcidoidea) is a generalist gregarious parasitoid of large dipteran pupae (including Sarcophagidae and Calliphoridae: Whiting, 1967; Werren *et al.*, 2010). Depending on the host species and whether or not a host has been already parasitized, females can oviposit more than 50 eggs on an individual host. Eggs are laid between the puparium wall and the host pupa, with larvae hatching and attaching themselves to the fly pupa to feed. On the blowfly *Calliphora vomitoria* egg-to-adult development takes approximately 14 days at 25 °C, with males eclosing and emerging from the host puparium before females. *Nasonia vitripennis* males are brachypterous and are unable to fly, remaining close to the emergence patch where they compete with each other for matings with emerging females. Females are fully winged and typically mate only once before dispersing to find new hosts. This life history creates extremely localized mating populations, selecting for female-biased sex ratios as envisaged by LMC theory (Hamilton, 1967; Taylor & Bulmer, 1980; Herre, 1985; Frank, 1986). LMC theory predicts that sex ratios should become increasingly female biased as LMC among related males increases. The extent of LMC will depend on how many females (or ‘foundresses’) contribute eggs to a patch, specifically in terms of the relative clutch sizes of the different foundresses (Werren, 1980; Stubblefield & Seger, 1990). The most intense LMC, and the most female-biased sex ratios, occurs if only one female oviposits on a patch. Repeated tests of *N*. *vitripennis* have generally confirmed predictions from both general and specific models of LMC in the laboratory (Werren, 1980, 1983; Orzack & Parker, 1986; Flanagan *et al.*, 1998; Shuker & West, 2004; Shuker *et al.*, 2005, 2006, 2007; West *et al.*, 2005) and in the wild (Molbo & Parker, 1996; Burton-Chellew *et al.*, 2008; Grillenberger *et al.*, 2008).

### Experimental strains

We used two iso-female *N*. *vitripennis* strains that were previously collected from a single population (De Hoge Veluwe, the Netherlands). The low sex ratio strain C222a (denoted L) was collected in 2003 by L.W. Beukeboom, and the high sex ratio strain HV06 (denoted H) was collected in 2004 by T. Koevoets, M.N. Burton-Chellew and E.M. Sykes (for collection details see Burton-Chellew *et al.*, 2008 and Grillenberger *et al.*, 2008). These strains were chosen from a panel of strains that was previously screened for sex ratio variation on the basis of their different single foundress sex ratios behaviour (Table 1, Pannebakker *et al.*, 2008). Strains were maintained on *Calliphora vomitoria* pupae as hosts at 25 °C, 16L : 8D light conditions.

**Table 1:** The experimental set-up and sample sizes for the genotyping and phenotyping for sex ratio and clutch size. Two hundred and ninety-nine clonal sibships of recombinant males were generated across two sets of crosses. Recombinant F2 males were derived from virgin F1 females, and sibships were created by crossing these F2 males to females from the high and low sex ratio lines. The F3 clonal sibships of these crosses were then phenotyped for single foundress sex ratio and clutch size.

	Recombinant F2 males		
		
Cross	Genotyped	Phenotyped	F3 broods analysed
1	276	156	2057
2	143	143	1657
Total	419	299	3714

**Fig. 1:**
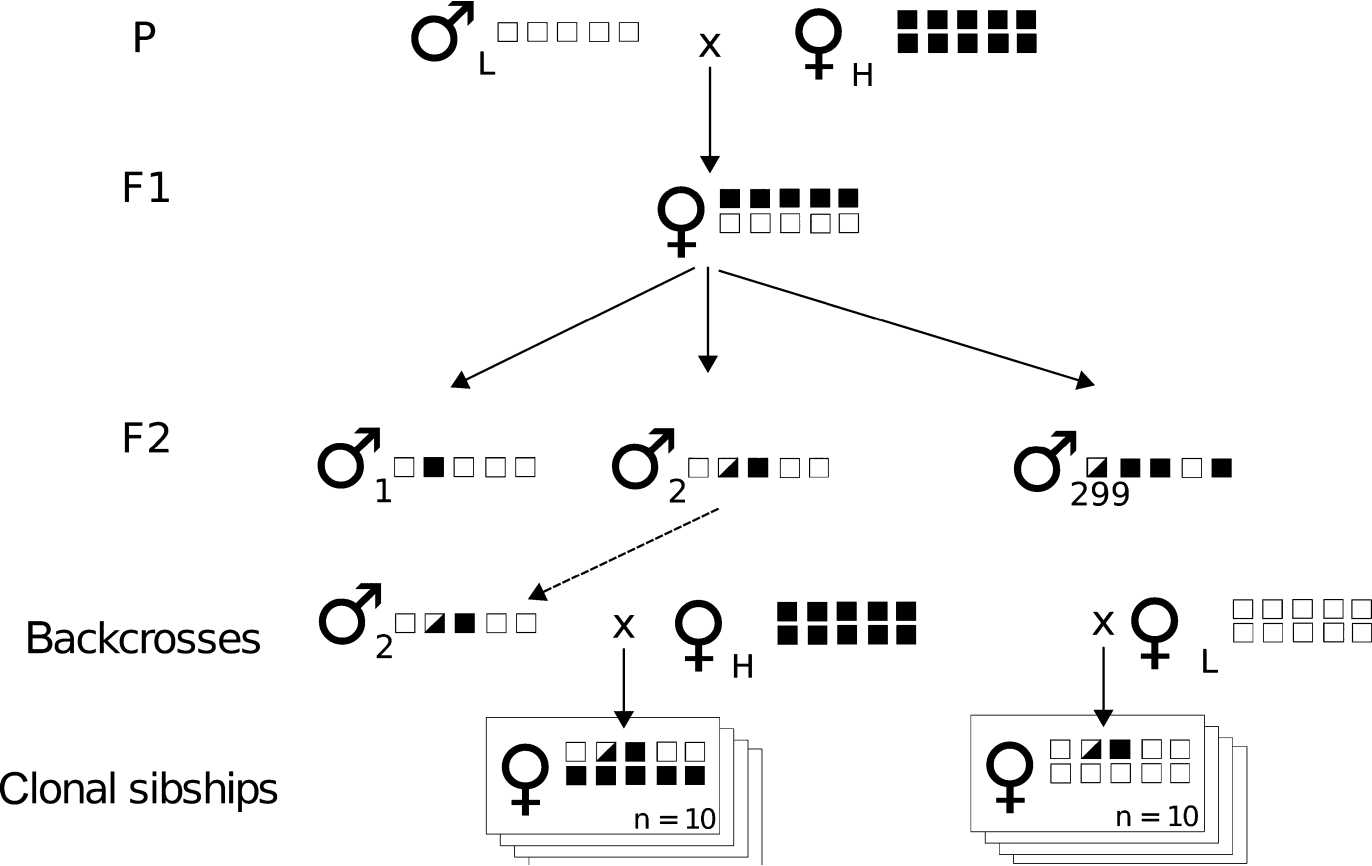
The experimental crossing procedure, used to generate clonal sibships. Virgin LxH F1 females produced recombinant F2 males, which were then backcrossed to both the H and the L parental line, to produce females in clonal sibships. These females were assayed for single foundress sex ratio and clutch size in each genetic background (*N* = 10 females per recombinant male genotype per background). In total, 299 F2 recombinant male genotypes from two sets of crosses were combined in a single quantitative trait locus analysis, with a further 120 male genotypes used for the linkage mapping. Squares represent the five different chromosomes of *Nasonia vitripennis.*

We took advantage of haplodiploid genetics to generate large numbers of genetically near-identical females with recombinant genotypes, following methods outlined in Velthuis *et al.* (2005). An L male was crossed to a virgin H female (generation P) to produce genetically identical F1 hybrid females (Fig. 1). The F1 females were allowed to oviposit as virgins, generating F2 males with recombinant genotypes between the L and H lines. Because *Nasonia* males are haploid, they produce genetically identical sperm. Each F2 male was then backcrossed to females from both the H and L strains, thus generating so-called clonal sibships. In two separate experimental blocks, we generated clonal sibships in both H and L backgrounds from a total of 299 recombinant males (Table 1). This set-up takes advantages of haplodiploid genetics in two ways: (i) it allows for replicate phenotyping of each recombinant haploid genotype expressed in both H and L backgrounds; (ii) it allows for the estimation of additive and dominance effects.

### Phenotyping

For each of the sibships, we isolated approximately ten 2-day mated old females in glass vials and provided them with a single host for 24 h as pretreatment to facilitate egg development. We then discarded the pretreatment hosts and gave each female a piece of chromatography paper soaked in honey solution for a further 24 h. We then provided these females with a single host for oviposition. After 3 h, we placed one-way escape tubes on the glass vials to allow females to disperse away from the patches, to limit superparasitism (Godfray, 1994; Shuker *et al.*, 2005). After 24 h, we removed the females and incubated the parasitized hosts at 25 °C. We randomly distributed these vials across racks to ensure that hosts from the same sibship were distributed across racks and shelves in the incubator. We allowed the resulting adult wasps to emerge and die prior to counting and sexing the brood, resulting in a total of 3714 clutches comprising 106 646 wasps (Table 1).

### Genotyping

We determined the genotypes of the L and H parental strains and of a total of 419 F2 recombinant males (Table 1). DNA from adult males was extracted using 5% Chelex 100 solution (Bio-Rad, Hercules, CA, USA). For genotyping, we chose 48 markers from the collection of microsatellite markers derived *in silico* from the *Nasonia* genome sequence (Release 1.0) by Pannebakker *et al.* (2010). We performed PCRs of microsatellite markers (Appendix 1) in a 10.0 μL volume (1× NH_4_ Buffer, 10 mm MgCl_2_, 1 mm dNTP mix, 4 mm of each primer, 0.5 units BIOTAQ DNA polymerase [Bioline Ltd, London, UK), 2 μL DNA (20 ng)]. The PCR temperature profile started with 4-min denaturation step at 94 °C followed by 39 cycles of 30 s at 94 °C, 30 s at the annealing temperature (*T*_a_) specified in Appendix 1, and 1 min at 72 °C, followed by 10 min at 72 °C. We determined length differences of the PCR products on 4% agarose gels stained with EtBr.

We generated an intra-specific *N*. *vitripennis* linkage map using a population of 419 recombinant F2 males, scored for 48 microsatellite markers. We used MapDisto software (version 1.7; Lorieux, 2007) to determine linkage and evaluate the order of markers on each of the five linkage groups. Marker order was confirmed using the *N*. *vitripennis* genome sequence (Werren *et al.*, 2010). We used Kosambi’s mapping function (Kosambi, 1944) to translate recombination fractions into map distances (cM). We visualized linkage groups using MapChart (version 2.1; Voorrips, 2002). The map generated in this study is an extension of the intra-specific *N*. *vitripennis* map presented in Beukeboom *et al.* (2010).

### QTL analysis

We identified QTL using Haley–Knott regressions (Haley & Knott, 1992) in the r language (version 2.9.0, Ihaka & Gentleman, 1996). We performed multiple regressions using a general linear mixed-effect model implemented using the ‘lme’ function in the nlme package (Pinheiro & Bates, 2000). To obtain the minimal adequate model, we first fitted the maximal model, containing sibship background (H/L), Parental Female ID and their interaction as fixed effects and F2 recombinant genotype as a random effect. Model simplification allowed us to drop Parental Female ID and the interaction between sibship background and Parental Female ID as a fixed effects (likelihood-ratio test; sibship background : Parental Female ID, 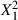 = 0.38, *P* = 0.54; Parental Female ID, 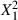 = 0.01, *P* = 0.91). Next, for each F2 male, we calculated the probabilities of the two alternative genotypes at every centiMorgan position along the chromosomes, conditional on the available marker data, using the R/QTL package (Broman *et al.*, 2003). R/QTL uses a hidden Markov model to calculate the probabilities of the true underlying genotypes given the observed multipoint marker data, with allowance for genotyping errors. Next, we tested for an association between sex ratio and genotype at each centiMorgan location using multiple regression. Sex ratios were calculated as the proportion of offspring that are male, and we arcsine-square-root transformed the data to remove the dependency of the variance on the mean. We regressed the transformed data on the conditional probability of a F2 male carrying the high sex ratio line (H) allele. The strength of the association between genotype at a location and transformed sex ratio was described as an *F*-statistic. We tested the association between clutch size and genotype in a similar way, where clutch size was transformed using sqrt(clutch size + 0.5).

We estimated significance thresholds for the association between marker genotype and phenotype by permutation (Churchill & Doerge, 1994). The phenotype data were permuted over the F2 genotypes, and the maximum *F*-statistic across all locations was recorded. One thousand permutations were performed to generate a null distribution of the *F*-statistic and the following thresholds obtained: the genome-wide significance threshold (*P* = 0.05) and chromosome-wide significance threshold (*P* = 0.05) levels as suggested by Lander & Kruglyak (1995). We determined the confidence intervals on the QTL locations by bootstrapping, for which we repeated the analysis on 1000 different data sets generated by re-sampling the F2 genotypes and associated phenotypes with replacement. We determined the location of the maximum *F*-statistic in each round of bootstrapping and obtained empirical 95% confidence intervals from the resulting distribution (Visscher *et al.*, 1996). We determined the percentage phenotypic variance explained as 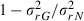, where 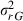 is the residual variance in the model with all genetic terms, and 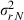 is the residual variance in a null model in which the genetic terms are omitted (Borevitz *et al.*, 2002). The percentage genetic variance explained was determined by 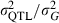, where 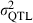 is the variance explained by the QTL 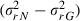, and 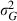 is the genetic variance in the F2 recombinant males. The effect of the QTL on sex ratio was determined by back-transforming the arsine-square-root transformed data obtained from the regression of each of the two genotypes for both backgrounds (H/L). Effects are expressed as (i) the difference between the two values, scaled to the mean sex ratio in that background (normalized QTL effect) and (ii) separated out as the genotypic additive (*a*) and dominance (*d*) effects. We did a similar calculation for the effect of QTLs for clutch size. We obtained the approximate confidence limits on QTL effects using the ‘intervals’ function of lme (Pinheiro & Bates, 2000) and back-transformed in a similar manner.

Evidence for pleiotropic QTLs for sex ratio and clutch size of QTLs was tested for by comparing the genome-wide test statistics for correlation (Keightley & Knott, 1999). Critical values were obtained by applying a Bonferroni correction for multiple comparisons with the equivalent number of independent tests determined from the QTL threshold *F*-statistics at *P* = 0.05.

### Broad-sense heritability

We estimated the broad-sense heritability *H*^2^ of sex ratio and clutch size in our data using linear mixed-effect models on the transformed data. F2 recombinant males were fitted as a random effect and variance components were estimated by REML. Linear mixed-effect models were implemented using the ‘lme’ function in the nlme package (Pinheiro & Bates, 2000) in the r language (ver. 2.10.1; Ihaka & Gentleman, 1996).

## Results

### Descriptive statistics

We analysed the sex ratios associated with recombinant F2 male genotypes produced by a cross between the low sex ratio line C222a (L) and the high sex ratio line HV06 (H) when then back-crossed into the H or L background. The recombinant genotypes produced intermediate sex ratios (Table 2), when measured in either the L or the H backgrounds. The recombinant F2 males showed a broad-sense heritability of *H*^2^ = 9.5% for sex ratio (likelihood-ratio test; 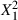 = 38.40, *P* < 0.0001), and *H*^2^ = 15.3% for clutch size (likelihood-ratio test; 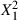 = 84.75, *P* < 0.0001). Our data showed a weak but significant positive correlation between sex ratio and brood size (binomial logistic regression: *b* = 0.005, 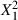 = 39.9, *P* < 0.0001).

**Table 2:** Descriptive statistics for the sex ratios (proportion male) and clutch sizes of *Nasonia vitripennis* lines C222A (L) and HV06 (H) From Pannebakker *et al.* (2008) and for the recombinant male genotypes from the cross C222A × HV06 when backcrossed into both the H and L backgrounds. Data are presented as means (standard error).

Line/cross	Sex ratio	Clutch size
C222A (L)	0.132 (0.008)	35.91 (1.75)
HV06 (H)	0.231 (0.027)	35.27 (1.80)
Recombinants (L)	0.151 (0.002)	29.16 (0.26)
Recombinants (H)	0.168 (0.003)	28.25 (0.27)

### Intra-specific linkage map

**Fig. 2:**
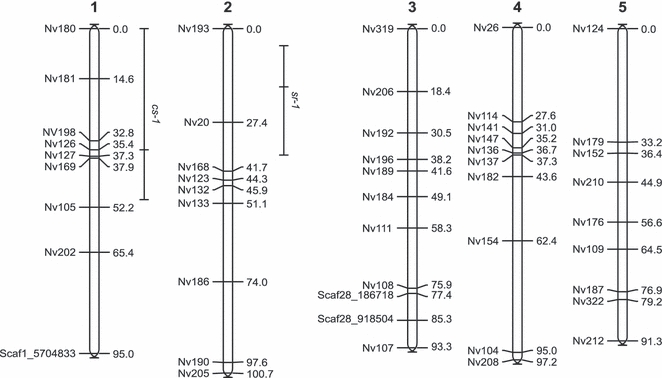
Intra-specific linkage map for *Nasonia vitripennis*. Map distances are calculated using Kosambi’s mapping function, with a total map length of 477.5 cM. The map is based on 419 recombinant F2 males genotyped for 48 microsatellite markers. Genome-wide significant quantitative trait locus (QTLs) for sex ratio (sr-1) and clutch size (cs-1) are indicated; the error bars represent 95% confidence interval for the QTL determined by bootstrapping.

Our linkage analysis resulted in a linkage map for *N. vitripennis* (Fig. 2) with five linkage groups (representing the five *N*. *vitripennis* chromosomes), spanning a total of 477.5 cM, and with an average marker distance of 11.11 cM.

### QTL for sex ratio and clutch size

**Fig. 3:**
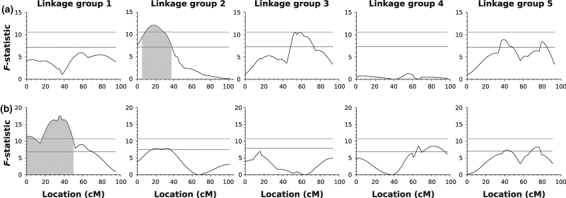
Locations of (a) sex ratio and (b) clutch size quantitative trait locus (QTL) in *Nasonia vitripennis*. *F*-statistic obtained by regressing genotype against sex ratio or clutch size. The grey, solid line is the 5% genome-wide significance threshold from permutation tests, and the black, dashed line is the 5% chromosome-wide significance threshold. The shaded region is the 95% confidence interval for the two QTL significant at the genome-wide level (obtained by bootstrapping; see main text for details).

There was one QTL for sex ratio significant at the genome-wide level (*P*<0.05) located on linkage group 2 at 17.0 cM (*F*_1,297_ = 12.12; Fig. 3). This QTL was associated with a change in sex ratio of 15% in the high background and 6% in the low background (Table 3). The low allele showed partial dominance over the high allele (degree of dominance *d/a* = −0.5). As expected from the low heritability of sex ratio (*H*^2^ = 9.5%), the QTL explained only 0.16% of the phenotypic variance, and 1.56% of the genetic variance in our cross-population. In addition, three further suggestive sex ratio QTL (significant at the chromosome-wide level, *P*<0.05) were identified on linkage group 3 (at 53.0 cM), and on linkage group 5 (at 39.0 cM and at 79.2 cM; Table 4). The QTL on chromosome 3 is particularly close to genome-wide significance (Fig. 3).

**Table 3:** Summary of genome-wide significant QTL identified for sex ratio and brood size in *Nasonia vitripennis*. CIs are confidence intervals.

			Variance explained (%)		Background					
									
Trait	Chr*	Location† (95% CI)	Phenotypic	Genetic		QTL effect‡ (95% CI)	Normalized QTL effect§	μ¶	a**	*d*††
Sex ratio	2	17.0 (5.0–37.0)	0.16	1.56	High	0.0251 (0.0249 to 0.0253)	+14.9%	0.168	0.016	−0.008
					Low	0.0083 (0.0078 to 0.0089)	+5.5%	0.136		
Brood size	1	35.4 (0.0–50.0)	0.13	0.76	High	−2.27 (−3.64 to −0.90)	−8.0%	25.92	−1.588	−0.798
					Low	−2.39 (−3.74 to −1.02)	−8.2%	29.09		

QTL, quantitative trait locus.

* Chromosome.

† Location in centiMorgans.

‡ Estimated effect size of the QTL, calculated as the difference in sex ratio (or brood size) between sibships inheriting the high vs. the low allele from the recombinant F2 male.

§ Change relative to the mean in the high and low genetic backgrounds.

¶ Estimated genotypic mean in the high and low backgrounds.

** Estimated additive effect of the QTL in the sibships. Positive values indicate that the H allele (from the high sex ratio line) confers greater sex ratio/brood size.

†† Estimated dominance effect of the QTL in the sibships.

**Table 4:** Summary of QTL identified for sex ratio and clutch size in *Nasonia vitripennis*. The most likely genomic locations are given as position along the chromosome. Significant QTLs at the 5% genome-wide level are given in bold type, and significant QTLs at the 5% chromosome-wide level are given in italic. Locations were determined in individual single-QTL models, whilst effect sizes were determined by simultaneously fitting all chromosome-wide significant QTL in one multiple-QTL model.

			Effect‡		Normalized QTL effect§	
				
Trait	Chr*	Location†	High background	Low background	High background (%)	Low background (%)
Sex ratio	**2**	**17.0**	**0.0147**	**0.0141**	**+8.8**	**+9.4**
	*3*	*53.0*	*0.0133*	*0.0127*	*+7.9*	*+8.4*
	*5*	*39.0*	*−0.0089*	*−0.0086*	*−5.3*	*−5.7*
	*5*	*79.2*	*−0.0059*	*−0.0056*	*−3.5*	*−3.7*
Clutch size	**1**	**35.4**	**−2.16**	**−2.19**	**−7.6**	**−7.5**
	*2*	*21.0*	*1.77*	*1.80*	*+6.2*	*+6.2*
	*2*	*32.0*	*0.06*	*0.06*	*+0.2*	*+0.2*
	*4*	*66.2*	*0.83*	*0.84*	*+2.9*	*+2.9*
	*4*	*83.0*	*1.30*	*1.32*	*+4.6*	*+4.5*
	*5*	*43.0*	−*1.05*	−*1.07*	−*3.7*	−*3.7*
	*5*	*76.0*	−*0.53*	−*0.54*	−*1.9*	−*1.9*

QTL, quantitative trait locus.

* Chromosome.

† Location in centiMorgans.

‡ Estimated effect size of the QTL, calculated as the difference in sex ratio (or brood size) between sibships inheriting the high vs. the low allele from the recombinant F2 male.

§ Change relative to the mean in the high and low genetic backgrounds.

There was one QTL for clutch size significant at the genome-wide level (*P*<0.05). This QTL was located on linkage group 1 at 35.4 cM (at marker Nv126: *F*_1,297_ = 17.65; Fig. 3) and was associated with a reduction in clutch size of 8% in both high and low backgrounds (Table 3). The high allele showed partial dominance over the low allele (*d/a* = 0.5). This QTL explained 0.13% of the phenotypic variance in clutch size, and 0.76% of the genetic variance. At the weaker chromosome-wide significance level, six further suggestive QTL were identified (Table 4), located on linkage group 2 at 21.0 and 32.0 cM, on linkage group 4 at 66.2 and 83.0 cM and on linkage group 5 at 43.0 and 76.0 cM. When considered together to test for pleiotropy, the genome-wide test statistics for sex ratio and clutch size did not reveal a significant correlation (*r* = 0.06, Pearson’s product–moment correlation, *t*_522_ = 1.44, *P* = 0.15).

Because additional QTL could only be found that were significant on a chromosome-wide level, we refrained from a formal analysis of epistasis between the different loci. However, a preliminary exploration of possible interactions between the loci did not reveal significant interactions between the genome-wide QTL and the suggestive QTL (data not shown). A more complete analysis of the genetic architecture of sex ratio in *Nasonia* will have to await the identification of further loci.

## Discussion

Here, we have described the first QTL for sex ratio variation in any organism. Differences in single foundress sex ratio between two wild-caught strains from the same population of *N. vitripennis* are associated with a region near the end of chromosome 2. Additional, weaker QTLs map to chromosomes 3 and 5. Intra-specific genetic variation in sex ratio in *N*. *vitripennis* is limited (Pannebakker *et al.*, 2008), and we required a large sample size to reveal these putative QTL (299 recombinants assayed in two backgrounds, using more than 3700 test females). However, we have been able to identify with confidence one QTL segregating in a natural population of wasps, with reasonable confidence in another. In addition, we have identified a genome-wide QTL influencing clutch size, as well as a number of lesser putative QTL. We have less idea of the underlying heritability of clutch size, and the two strains used were not chosen to maximize differences in clutch size, but our experiment was still able to resolve an intra-population QTL for this important life-history trait.

The QTL identified at the 5% genome-wide level span a large part of the genome, i.e. 32 cM for sex ratio variation corresponding to ∼21.3 Mb, and 50 cM for clutch size variation corresponding to ∼33.3 Mb assuming a genome-wide recombination rate of 1.5 cM Mb^−1^ (Niehuis *et al.*, 2010). These figures correspond to approximately 1257 candidate genes for sex ratio and 1965 for clutch size, assuming ∼59 genes per Mb (Niehuis *et al.*, 2010). Our results provide the starting point in the search for possible candidate loci for sex ratio and brood size, but given the size of these genomic regions and the number of possible candidates, future studies will require a much higher resolution before we can start to screen the genome for candidate loci. Alternatively, a combined approach taking advantage of studies of the genes expressed during oviposition and sex allocation decision-making may help generate candidate genes in the QTL regions of interest; we are currently attempting this.

Our weaker sex ratio QTL on chromosome 3 is close to genome-wide significance, but the QTL suggested for chromosome 5 are only convincingly significant at the chromosome-wide threshold level. However, recent work exploring inter-specific differences in sex ratio among the *Nasonia* genus has identified a region on chromosome 5 that influences sex ratio when introgressed from *Nasonia giraulti* into *N*. *vitripennis* (Werren *et al.*, 2010). This region includes the well-known red eye mutant locus *STDR*. Whilst neither of our QTL is centred on this region, our second QTL (centred on 79 cM) is close. This raises the possibility that genes associated with differences in sex ratio between *Nasonia* species are also segregating within populations, unlike the case for courtship song differences within and between *Drosophila* species (Gleason & Ritchie, 2004; see also Arbuthnott, 2009). The differences between the *Nasonia* species in single foundress sex ratios are thought to be arising from differences in the extent of so-called within-host mating (mating that occurs before males and females emerge from the host puparium: Drapeau & Werren, 1999). Within-host mating leads to very extreme LMC if all the brood are relatives, and *N*. *giraulti* displays the greatest extent of within-host mating, and *N*. *vitripennis* the least (see also Leonard & Boake, 2006). As such, it would also be useful to identify genome regions associated with differences between the species in within-host mating, and see whether they co-occurred with putative sex ratio QTL.

Even though we found one single genome-wide significant QTL for sex ratio, it only explains a small part of the phenotypic (0.16%) and genetic variance (1.56%). This suggests that many other genes are segregating for sex ratio. In line with this, we found several minor QTL segregating for sex ratio, and our estimate of QTL number is likely to be an underestimate (Beavis, 1994). A genetic architecture of multiple loci underlying sex ratio is compatible with recent theoretical predictions from mutation accumulation experiments. Based on estimates of mutational parameters and on the natural variation for sex ratio, Pannebakker *et al.* (2008) predicted that the genetic variation for sex ratio in *Nasonia* is maintained by selection on pleiotropic loci with effects on other fitness-related traits. Pleiotropy as the primary cause of genetic variation in sex ratio is more plausible when more loci are involved (Barton & Turelli, 1989).

Our data allow a weak test of the prediction of Pannebakker *et al.* (2008). Here, we of course have only considered one other fitness-related trait: clutch size. The genome-wide QTL for clutch size and sex ratio were clearly independent of each other, occurring on different chromosomes. There was also no hint of a QTL for sex ratio on chromosome 1 that corresponded to the clutch size QTL (Fig. 3). When tested for concordance between the test statistics of sex ratio and clutch size as an indication for the existence of pleiotropic QTL, we could not detect a significant correlation. There was, however, a weakly suggestive QTL for clutch size on chromosome 2 that overlapped with our major sex ratio region. In addition, the weak QTL for sex ratio and clutch size located on chromosome 5 potentially overlap. That said these particular QTL are at best preliminary and of course cover very many possible candidate loci; as such the conclusion we draw at the moment is that loci pleiotropic for clutch size and sex ratio remain possible. A more detailed multitrait QTL analysis should be carried out before pleiotropy can be formally excluded. Of course, analysis of different fitness-related traits may yet throw up other associations with sex ratio QTL, and various aspects of *Nasonia* behaviour and life history are currently the focus of genetic study (Werren *et al.*, 2010).

The genetic basis of sex ratio is of prime importance if we want to understand sex ratio evolution. The number of loci and alleles underlying the trait will influence its dynamics in populations (Basolo, 1994). Even though the present study offers a first insight, we are currently only at the start of uncovering the genetic basis of sex ratio in *Nasonia*. Our limited knowledge of the genetic basis of sex ratio contrasts with our increasingly sophisticated knowledge of how sex is determined, both in *Nasonia* (Verhulst *et al.*, 2010; Werren *et al.*, 2010) and across other taxa more generally (Bull, 1983). We now have a very good idea of key aspects in the sex determination cascade, and which components tend to vary across species and which tend to be conserved (Sanchez, 2008). The extent to which behavioural sex allocation mechanisms interact with the underlying machinery is unknown and they may well be totally separate. However, the increasing awareness that there may be genomic conflicts over sex allocation has meant that a renewed focus has been placed on how and when sex is determined, and what genetic entities (such as males and females) may be trying to influence it (e.g. Shuker *et al.*, 2007, 2009; Uller *et al.*, 2007; Wild & West, 2009). As such, sex ratio control and sex determination may be a joint evolutionary battleground worth considering together (Werren & Beukeboom, 1998).

The success of phenotypic models of sex allocation could be taken to suggest that genetic constraints are unimportant in the evolution of adaptive sex ratios, but that same success has perhaps kept focus away from the genetics underlying sex ratio. Perhaps just as important has been the apparent contradiction between chromosomal sex determination and adaptive sex allocation, especially in vertebrates (e.g. what is the mechanism?), or the low heritability often associated with sex ratio (as seen in *Nasonia*). However, by identifying QTL in a trait that typically has low heritability within populations, we can now begin to isolate regions of interest for further study. Combined with insights from inter-specific differences between the four different *Nasonia* species in sex ratio, and potentially any other traits genetically associated with sex ratio (Werren *et al.*, 2010), we can also begin to explore the genetic architecture of sex ratio. Whilst we may perhaps not expect to find genetic constraints *per se* to sex ratio adaptation in *Nasonia* (female *Nasonia* are rather good at solving sex ratio problems after all), we can turn the question around and ask what the genetic architecture of a well-adapted phenotypic trait actually looks like. We may then begin to consider how natural selection shapes the genome from the phenotype inwards, rather than from the genotype outwards. Given the scepticism about the role of natural selection in shaping aspects of the genome more generally (e.g. Lynch, 2007), it may prove useful to let well-understood phenotypes guide us to the evolutionary processes occurring at the molecular level, in addition to considering sequence evolution itself.

## Supporting information

**Appendix S1** Microsatellite markers.

As a service to our authors and readers, this journal provides supporting information supplied by the authors. Such materials are peer-reviewed and may be recognized for online delivey, but are not copy-edited or typeset. Technical support issues arising from supporting information (other than missing files) should be addressed to the authors.
