# Supplementary material for: The quantitative genetic basis of sex ratio variation in Nasonia vitripennis: a QTL study
Source: J Evol Biol. 2011 Jan;24(1):12–22. doi: 10.1111/j.1420-9101.2010.02129.x (PMC3025119; doi:10.1111/j.1420-9101.2010.02129.x)
Supplement: Supplementary file 1 [file jeb0024-0012-SD1.doc]

**Appendix S1** Microsatellite markers. Microsatellite markers developed from the *Nasonia* genome assembly (1.0). Information on marker location was obtained from an in-silico screen by Pannebakker *et al.* (2010) of the *N*. *vitripennis* trace archive sequences (Werren *et al.*, 2010). Chromosomal location, repeat sequence, primer sequences, annealing temperatures, and Genbank accession numbers are given. The amplicon lengths were directly calculated from the *N. vitripennis* sequence by the program msatfinder (for details see Pannebakker *et al.*, 2010).

| **Locus** | **Chromosome^1^** | **Repeat sequence** | **Amplicon length *N.vitripennis*** | **Primer sequence** | **Annealing temperature** | **Genbank accession no.** | **Reference** |
| --- | --- | --- | --- | --- | --- | --- | --- |
| Nv20 | 2 | GT | 105 | 5' TGACGAAGTATCCGAGAAG 3' 5' TCGAAAAACGATATTGCTCG 3' | 58 | AY262039 | Pietsch *et al.*, 2004 |
| Nv26 | 4 | GT | 142 | 5' TTCGCAGCTTTCCTTTGC 3' 5' AGCAGCTAGTATGAACC 3' | 58 | AY262047 | Pietsch *et al.*, 2004 |
| Nv104 | 4 | ct | 212 | 5' aagcgtccgtaaaagtctgc 3’ 5' gatttcccggagcttcctac 3' | 64 | FJ156110 | Beukeboom *et al.*, 2010 |
| Nv105 | 1 | ta | 201 | 5' CGAGTTACACACGAGGAGCA 3’ 5' CTCTACTCGCTGCGCACAT 3' | 60 | FJ156111 | Beukeboom *et al.*, 2010 |
| Nv107 | 3 | tc | 201 | 5' TCGTGGCACGTAGGCTACTA 3’ 5' CAGTCGGCAACAACAGATTG 3' | 65 | FJ156112 | Beukeboom *et al.*, 2010 |
| Nv108 | 3 | ga | 188 | 5' CTGTGCAGGACTTTGACGAA 3’ 5' ACACTCAATCCCTCGTGCTT 3' | 60 | FJ156113 | Beukeboom *et al.*, 2010 |
| Nv109 | 5 | ag | 196 | 5' acgacgacggtggagagtag 3’ 5' gcttactctcgggaactgga 3' | 65 | FJ156114 | Beukeboom *et al.*, 2010 |
| Nv111 | 3 | tc | 199 | 5' catcgaaatcgcgttactca 3’ 5' ctttggtttcgcttctgctc 3' | 65 | FJ156115 | Beukeboom *et al.*, 2010 |
| Nv114 | 4 | GA | 204 | 5' ATG GGC AAT AAA ACG AAA CG 3' 5' CAT CCT TGC GGA GAC ACT AA 3' | 60 | FJ156231 | Beukeboom *et al.*, 2010 |
| Nv123 | 2 | tc | 192 | 5' CGCTACTCGTGCGCTATTATT 3’ 5' CCCAAAAGCACTTTGAGTCC 3' | 60 | FJ156124 | Beukeboom *et al.*, 2010 |
| Nv124 | 5 | ga | 194 | 5' CAACACTGATTCGTGCATCC 3’ 5' GAACTCTGAACCACGGCAAT 3' | 65 | FJ156125 | Beukeboom *et al.*, 2010 |
| Nv126 | 1 | ag | 208 | 5' CCACCAGGAGCTTCAAAAAG 3’ 5' CATCGCCACACTCTCACACT 3' | 60 | FJ156127 | Beukeboom *et al.*, 2010 |
| Nv127 | 1 | at | 226 | 5' GACTGCATCCGTCCATTGAT 3’ 5' ATAAGAGCGCGGTGGAATAA 3' | 60 | FJ156128 | Beukeboom *et al.*, 2010 |
| Nv132 | 2 | at | 254 | 5' TAGGGGATCCTGAATTTCCA 3’ 5' CTCTTTGGGAATGGAAAACG 3' | 61 | FJ156131 | Beukeboom *et al.*, 2010 |
| Nv133 | 2 | ta | 235 | 5' GACGTTAGATCTCTGTCGCTTC 3’ 5' ACGACAAAATTTCGCCTACG 3' | 60 | FJ156132 | Beukeboom *et al.*, 2010 |
| Nv136 | 4 | ag | 197 | 5' ACGTCGAGTTGCTTTGCTTT 3’ 5' ACTTTCGCATCCACACTTCC 3' | 60 | FJ156133 | Beukeboom *et al.*, 2010 |
| Nv137 | 4 | ga | 202 | 5' GGAAATTTGGCAAACCTCAA 3’ 5' CGTGTTTGTTTTTGCCACTG 3' | 60 | FJ156134 | Beukeboom *et al.*, 2010 |
| Nv141 | 4 | ta | 199 | 5' CGATGTACTTTGCGAACGTG 3’ 5' CAGCTTTTCTTCAACGCACA 3' | 61 | FJ156137 | Beukeboom *et al.*, 2010 |
| Nv147 | 4 | GA | 216 | 5' CCCCGACTTGATTAGCAAAA 3’ 5' TTAGCCAATTAACGCGATGAC 3' | 60 | FJ156141 | Beukeboom *et al.*, 2010 |
| Nv152 | 5 | ga | 207 | 5' TTCCCTCTGCGAGGCTATTA 3’ 5' AGGCCAAAAGTGCACAAAGT 3' | 60 | FJ156145 | Beukeboom *et al.*, 2010 |
| Nv154 | 4 | tc | 200 | 5' TATCGATCTCGAGCCTCAGC 3’ 5' ACACTTTTTGCGCGTTTTCT 3' | 60 | FJ156147 | Beukeboom *et al.*, 2010 |
| Nv168 | 2 | TA | 215 | 5' CACCACACACACCCTCTCAC 3’ 5' TAAGCGCTCACATCCAGTTG 3' | 60 | FJ156161 | Beukeboom *et al.*, 2010 |
| Nv169 | 1 | ct | 217 | 5' GCCCTTTAACCAAGCACTGA 3’ 5' GGCTCATTCCAGTGAAGGAC 3' | 60 | FJ156162 | Beukeboom *et al.*, 2010 |
| Nv176 | 5 | ga | 209 | 5' ATCCGTCTCCTCGTCCTTTT 3’ 5' GGCACGGCTCAATAAAGAAG 3' | 58 | FJ156169 | Beukeboom *et al.*, 2010 |
| Nv179 | 5 | ag | 200 | 5' GCTTATACACGGGCGAATGT 3’ 5' AGCCGGTGAATAGCATTCTG 3' | 58 | FJ156172 | Beukeboom *et al.*, 2010 |
| Nv180 | 1 | AG | 209 | 5' ATAAGCCGCCGAAAAGAGAG 3’  5' TCCAAAGTGTGCGATACTGC 3' | 58 | FJ156173 | Beukeboom *et al.*, 2010 |
| Nv181 | 1 | ga | 206 | 5' TTTTCGCGATTTTCTGGAAC 3’ 5' ATTGGTTACTCGCGGCTGTA 3' | 58 | FJ156174 | Beukeboom *et al.*, 2010 |
| Nv182 | 4 | ta | 203 | 5' ACGGCGAAGAGCATCTTAAA 3’ 5' GCAGCGTGTGACAAAGAAGA 3' | 58 | FJ156175 | Beukeboom *et al.*, 2010 |
| Nv184 | 3 | tc | 209 | 5' GCGTCATCGATGCATTTCTT 3’ 5' TCTCGGGAGAGATTCAGTACG 3' | 58 | FJ156177 | Beukeboom *et al.*, 2010 |
| Nv186 | 2 | ag | 179 | 5' GAATCGCTGCAGTTGCCTAT 3’ 5' CACGATGCTTGCGTATATGTG 3' | 58 | FJ156178 | Beukeboom *et al.*, 2010 |
| Nv187 | 5 | ct | 199 | 5' GCGGACGACTCAGACTCATT 3’ 5' GCAATTACGCGGTGAATAGA 3' | 60 | FJ156179 | Beukeboom *et al.*, 2010 |
| Nv189 | 3 | ga | 202 | 5' CTACTGCGATCTGACGCTTG 3’ 5' GTGGAAAAATATCGCGCTTC 3' | 58 | FJ156181 | Beukeboom *et al.*, 2010 |
| Nv190 | 2 | ta | 183 | 5' CAGGTGCTCCTGTGACAAAT 3’ 5' CGCGGAAGCAAAAGACTACT 3' | 58 | FJ156182 | Beukeboom *et al.*, 2010 |
| Nv192 | 3 | ga | 206 | 5' TGCCTGCTATAGTCGTTGGA 3’ 5' TGTGTGTGTGTACTCGGCTTC 3' | 58 | FJ156184 | Beukeboom *et al.*, 2010 |
| Nv193 | 2 | at | 200 | 5' CTTTGTGCAAACGTCAGTCG 3’ 5' CGGCTCGCGTTAGAAAGTTA 3' | 58 | FJ156185 | Beukeboom *et al.*, 2010 |
| Nv196 | 3 | GA | 213 | 5’ AATGTTTCATCGCAGCACAC 3’ 5’ AGAGCGCGAGAGCTACAGAG 3’ | 58 | FJ156186 | This study |
| Nv198 | 1 | TA | 200 | 5’ AAAAACGGAGACGACGAAGA 3’ 5’ TCCGTCGAATGTGAATAATCC 3’ | 64 | FJ156188 | This study |
| Nv202 | 1 | TC | 209 | 5' TAATTCCCGAATGCGTCCTA 3’ 5’ GGGGGAGAGGGTATGTAAGG 3’ | 58 | FJ156192 | This study |
| Nv205 | 2 | AG | 187 | 5’ AATGCTCAAGCCAACTCGAT 3’ 5’ CGACATCACGACTCATCCAA 3’ | 58 | FJ156195 | This study |
| Nv206 | 3 | TC | 197 | 5' CGGGGAGTTGAAAATGAAAA 3' 5' TATATAACCGCGGACGAAGC 3' | 58 | FJ156196 | This study |
| Nv208 | 4 | TA | 236 | 5' TCGAAGGTTGAGAAAAAGACATC 3' 5' CGTCTACATGCTGCACACAG 3' | 58 | FJ156198 | This study |
| Nv210 | 5 | AG | 207 | 5’ TTCACTCGCGACTTATACGC 3’ 5’ GTAAGGCCATCGAGCAAGAG 3’ | 58 | FJ156200 | This study |
| Nv212 | 5 | AG | 200 | 5’ GTTTTAAGGCTTGCGGCTTT 3’ 5’ GTTGCCAGTCGCGAAATTAT3’ | 58 | FJ156202 | This study |
| Nv319 | 3 | CT | 220 | 5' TTTGAGGTTATGCGTCGTTTC 3' 5' GAGCGGAGTGCTTCATTCAG 3' | 60 | FJ156222 | Beukeboom *et al.*, 2010 |
| Nv322 | 5 | GT | 201 | 5' CGAAAGAAGCCAAGCATAGAA 3' 5' GAGAAAAATCGGGTCGAAGT 3' | 60 | FJ156225 | Beukeboom *et al.*, 2010 |
| Scaffold1_5704833 | 1 | CA | 133 | 5’ GCCATCCAGGAAACTCCTCT 3’ 5’ TTCGCCTCTCTTTCCCATAA 3; | 60 | - | O. Niehuis (unpublished data) |
| Scaffold28_186718 | 3 | TC | 151 | 5’ CTCCCCGCAACAATAACG 3’ 5’ GATATTGTGCGGCGCTTC 3’ | 60 | - | O. Niehuis (unpublished data) |
| Scaffold28_918504 | 3 | CCG | 236 | 5’CCGTGTGTGACGATGATGA 3’ 5’ TTGCTCAAACGCGAGTAAAA 3’ | 60 | - | O. Niehuis (unpublished data) |

^1^ Chromosome designation according to Rütten **et al.** (2004).

**References**

Beukeboom, L.W., Niehuis, O., Pannebakker, B.A., Koevoets, T., Gibson, J.D., Shuker, D.M. *et al.* 2010.. A comparison of recombination frequencies in intraspecific versus interspecific mapping populations of *Nasonia*. *Heredity* **104**: 302–309.

Pannebakker, B.A., Niehuis, O., Hedley, A., Gadau, J. & Shuker, D.M. 2010. The distribution of microsatellites in the *Nasonia* parasitoid wasp genome. *Insect Mol. Biol.* **19**(Suppl. 1): 91–98.

Pietsch, C., Rütten, K. & Gadau, J. 2004. Eleven microsatellite markers in Nasonia, Ashmead 1904 (Hymenoptera; Pteromalidae). *Molecular Ecology Notes* **4**: 43–45.

Rütten, K.B., Pietsch, C., Olek, K., Neusser, M., Beukeboom, L.W. & Gadau, J. 2004. Chromosomal anchoring of linkage groups and identification of wing size QTL using markers and FISH probes derived from microdissected chromosomes in *Nasonia* (Pteromalidae: Hymenoptera). *Cytogenetic and Genome Research* **105**: 126–133.

Werren, J.H., Richards, S., Desjardins, C.A., Niehuis, O., Gadau, J., Colbourne, J.K. *et al.* 2010. Functional and evolutionary insights from the genomes of three parasitoid *Nasonia* species. *Science* **327**: 343–348.
